# Design, Development, and Testing of Machine Learning Models to Estimate Properties of Friction Stir Welded Joints

**DOI:** 10.3390/ma18010094

**Published:** 2024-12-29

**Authors:** Sajjad Arif, Abdul Samad, Muhammed Muaz, Anwar Ulla Khan, Mohammad Ehtisham Khan, Wahid Ali, Farooque Ahmad

**Affiliations:** 1 Department of Mechanical Engineering, Aligarh Muslim University, Aligarh 202002, India; sajjad.aarif@gmail.com (S.A.); abdulsamadamuzhcet@gmail.com (A.S.); muhammedmuaz@zhcet.ac.in (M.M.); 2 Department of Electrical Engineering Technology, College of Applied Industrial Technology, Jazan University, Jazan 45142, Saudi Arabia; 3 Department of Chemical Engineering Technology, College of Applied Industrial Technology, Jazan University, Jazan 45142, Saudi Arabia; mekhan@jazanu.edu.sa (M.E.K.); wzali@jazanu.edu.sa (W.A.); 4 Department of Mechanical Engineering Technology, College of Applied Industrial Technology, Jazan University, Jazan 45142, Saudi Arabia; fahmad@jazanu.edu.sa

**Keywords:** welding, machine learning, ultimate tensile strength, hardness

## Abstract

This paper estimates friction stir welded joints’ ultimate tensile strength (UTS) and hardness using six supervised machine learning models (viz., linear regression, support vector regression, decision tree regression, random forest regression, K-nearest neighbour, and artificial neural network). Tool traverse speed, tool rotational speed, pin diameter, shoulder diameter, tool offset, and tool tilt are the six input parameters in the 200 datasets for training and testing the models. Deep learning artificial neural networks (ANN) exhibited the highest accuracy. Therefore, the ANN approach was used successfully to estimate the UTS and the hardness of friction stir welded joints. Additionally, the relationship of pin diameter, tool offset, and tool rotation speed over UTS and hardness were extracted over the collected data points. Furthermore, experimental results, such as UTS and hardness of steel–magnesium-based welded joints and model estimated results, were compared to cross-check model generalization capability. It was noted that ANN estimates and experimental results at desired processing conditions are consistent with sufficiently high accuracy.

## 1. Introduction

Welding is an essential manufacturing process for achieving components with complex configurations. However, it is considered a secondary step in manufacturing a product, followed by basic techniques, i.e., casting, machining, and forming. In most situations, we cannot produce a product as a single part; therefore, to obtain multi-functional products, the need for welding and its development has changed exponentially in the last few decades [1]. The use of composite materials is widespread in the automotive and aerospace industries due to their advantageous properties, including high specific strength, low densities, and the ability to reduce weight [2]. However, traditional fusion welding processes used to join different metals and alloys can result in poor weld zone characteristics in terms of strength and reliability. These challenges stem from excessive heat, differences in crystal structure, and variations in the physiochemical properties of the parent metals and alloys. As a result, coarse grains form in the weld zone, and numerous brittle intermetallic compounds are created [3].

Friction stir welding (FSW), a solid-state welding technique developed by Wayne Thomas in 1991 at The Welding Institute (TWI) in the U.K., can be utilized for welding various metals and alloys [4,5]. During FSW, the parent material does not melt because its processing temperature is lower than the material’s melting point. Eventually, the weld bead becomes free from unwanted metallurgical reactions. It is regarded as an environment-friendly and energy-efficient process. Further, FSW also displays vital catching features such as low distortions, minimal residual stresses, and no fumes and spatters. Nowadays, FSW is widely used for welding many low- and high-melting-point materials, such as aluminium alloys, magnesium alloys, and titanium and nickel [6,7,8].

Forming high-strength joints through FSW is essential for both academic interests and industrial applications. Increasing porosity and brittle intermetallic compounds decrease their static and dynamic behaviour to many folds. However, these issues can be minimized by understanding the complex relationship between various process parameters such as rotational speed, transverse speed, shoulder diameter, pin diameter, offset, tilt angle, etc. Many attempts have been made in both experimental and analytical directions for process optimization and confirming defect-free welds with improved metallurgical properties and mechanical behaviour.

### 1.1. Experimental Investigations

Zhao et al. [9] examined the impact of the pin profile (viz., column pin, taper pin, column screw thread pin, and taper screw thread pin) on the welded joint of Al2014 alloys. He found that the taper screw thread pin performed better in creating a strong bond. Bisadi et al. [10] studied the impact of different tool pin shapes (square, triangular and cylindrical) on the friction stir welding (FSW) of Al 7075 alloys. He inferred that workpiece temperature is more influenced by pin shoulder than pin diameter. The tool linear and rotation speeds are the most influential parameters in FSW, as they govern heat input and material flow. Kanemaru et al. [11] and Liu et al. [12] studied the influence of these parameters. They concluded that the strain region moves from the retreating side to the advancing side and widens the strained area. Fu et al. [13] studied the influence of rotation speed and welding speed on the FSW joints of dissimilar metals of 6061-T6 aluminium alloy to AZ31B magnesium alloy. The optimum set of welding and rotational speeds for higher strength were 30 and 60 mm/min and 600–800 rpm. Any departure with the welding and rotation speed leads to poor weld quality. Sahu et al. [14] studied the influence of tool offset on the morphology and structure of FSW joints of dissimilar materials. The authors concluded that a slight tool offset greatly influences intermetallic compounds’ material flow behaviour and volume. Watanabe et al. [15] concluded that maximum ultimate tensile strength could be achieved by providing a 0.2 mm offset. A higher offset reduces tensile strength by mixing large fragmented steel particles in the aluminium matrix.

### 1.2. Analytical and Statistical Investigations

Analytical and mathematical simulation is often used to model friction stir welding (FSW) due to its ability to accurately simulate heat generation, temperature distribution, material flow, and defect prediction. However, experimental investigation is costly, time-consuming, and limited in scope and cannot explain the complex interaction between process parameters and output results. Suresha et al. [16] conducted experiments with the L9 Taguchi orthogonal array to analyze the influence of tool pin profiles on the tensile strength of AA7075-T6 FSW joints. The percentage contributions of welding parameters, including tool rotational speed, traverse speed, and plunge depth, to tensile strength were analyzed using the signal-to-noise (S/N) ratio and analysis of variance (ANOVA). Javadi et al. [17] optimized the residual stresses of friction welded joints of 5086 aluminium plates through the statistical design of the experiment (DOE). ANOVA analysis concluded that feed rate is the most significant parameter for longitudinal residual stress. However, the shoulder and pin diameter effects were insignificant. Sarsilmaz et al. [18] conducted experiments according to a full factorial design to determine the influence of stirrer speed, traverse speed, and spindle rotational speed on UTS and the hardness of FSW joints of aluminium.

In recent years, the application of machine learning has increased tremendously in solving the complex behaviour of manufacturing processes. These trained algorithms can quickly learn previous data and provide information regarding the complex interrelationship between various processing parameters [19]. In the field of materials and manufacturing research, researchers have explored the use of artificial neural networks (ANNs) [20,21], the adaptive neuro-fuzzy inference system (ANFIS) [22,23], and regression analysis [24] to prioritize, plan, optimize, and forecast experimental results. This study introduces a novel application of machine learning models to predict the ultimate tensile strength (UTS) and hardness of friction stir welded joints by considering a comprehensive set of process parameters. While previous studies have focused on individual parameters or simple regression models, our work expands the scope by evaluating six different machine learning algorithms, with a particular focus on the artificial neural network (ANN) for accurate prediction. Additionally, this research incorporates a diverse dataset, including both similar and dissimilar material joints, which enhances the generalizability and practical applicability of the model across various welding scenarios.

After an extensive review of the literature, the following process parameters were identified as critical for friction stir welding (FSW) and were therefore selected for this study:

- Tool traverse speed and rotational speed: These are key determinants of heat generation and material flow, directly influencing weld quality, ultimate tensile strength (UTS), and hardness.
- Pin diameter and shoulder diameter: These geometrical features play a significant role in material stirring and mixing, impacting grain refinement, defect formation, and mechanical properties.
- Tool offset: Particularly important for dissimilar material welding, tool offset helps balance heat distribution, ensuring defect-free joints with improved strength.
- Tool tilt angle: This parameter aids in optimizing the plunge force and enhances material flow around the tool, reducing defects such as tunnelling.

These parameters were chosen based on their frequent citation and critical role in determining the mechanical and metallurgical properties of FSW joints, as highlighted in the reviewed studies.

This deficiency in the literature motivated the authors to design, develop, and test a machine learning model for the following reasons: (a) to establish the relationship between key process parameters of friction stir welding and (b) the developed model will be used to estimate the hardness and ultimate tensile strength of friction stir welded joints.

## 2. Modelling Procedure

**Figure 1:**
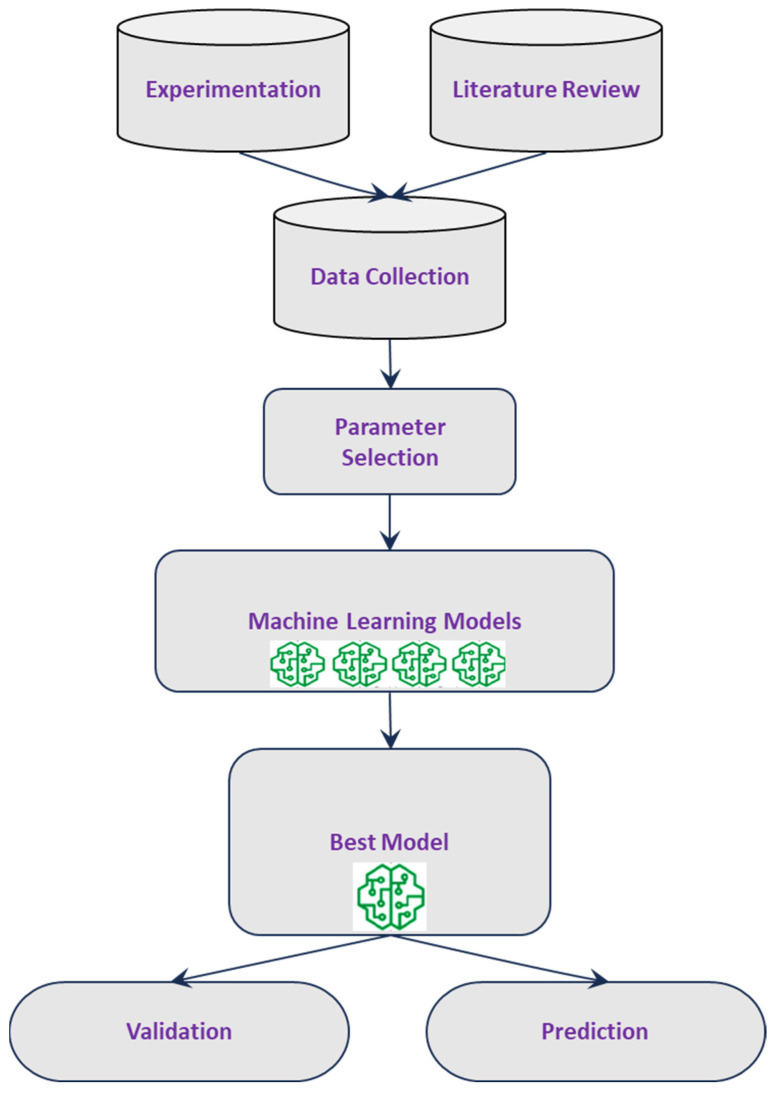
Procedure of machine learning modelling.

Creating machine learning models involves three main steps: data collection, model generation, and model validation. This process, illustrated in Figure 1, begins with data collection and concludes with model validation and output prediction. In this work, 200 data groups were collected and screened for machine learning models’ design, development, and testing, as depicted in Table 1.

### 2.1. Collection of Data

**Figure 2:**
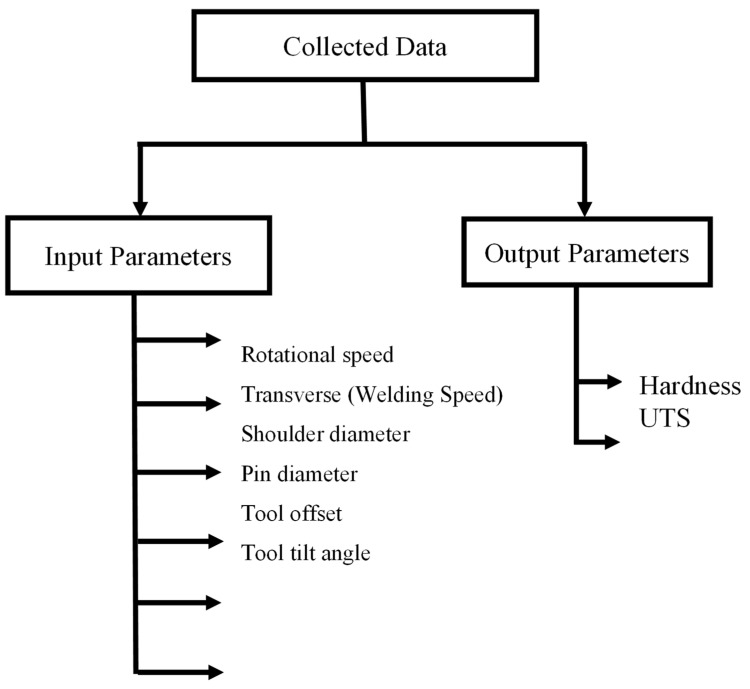
Various input and output parameters.

Data collection is one of the most fundamental steps before generating a machine learning model. The two main data sources are the author’s laboratory experiments and literature review [25,26,27,28,29,30,31,32,33,34,35,36,37,38,39,40]. The dataset, given in Table 1, used in this study consists of 200 data points collected from both similar and dissimilar material joints. Among these, approximately 50% represent similar material joints, such as aluminium–aluminium and steel–steel combinations, while the remaining 50% correspond to dissimilar material joints, including combinations such as aluminium–magnesium and steel–magnesium. This distribution ensures that the dataset encompasses a wide range of material combinations, allowing the models to generalize well across different welding scenarios. Figure 2 and Table 2 shows the various input and output parameters used in the present study.

### 2.2. Modelling

**Figure 3:**
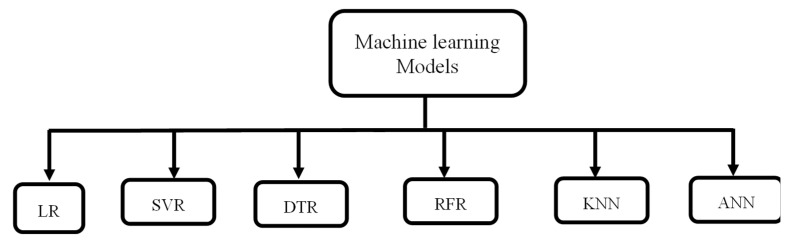
Different machine learning models used.

The present study primarily focused on six different and well-known machine learning algorithms to discover the best model. Figure 3 depicts the various machine learning models that were used in the present study.

We selected six machine learning models to thoroughly assess their predictive performance: LR, KNN, DT, SVR, ANN, and RF regression. LR predicts outputs based on a linear relationship between input and output variables, while KNN regression calculates the target variable by identifying a sample’s nearest neighbours using the Euclidean distance function (*d*), as shown in Equation (1):

(1) $$d=\sqrt{\sum_{i=1}^{n}{\left(x_{i}-y_{i}\right)}^{2}}$$

**Figure 4:**
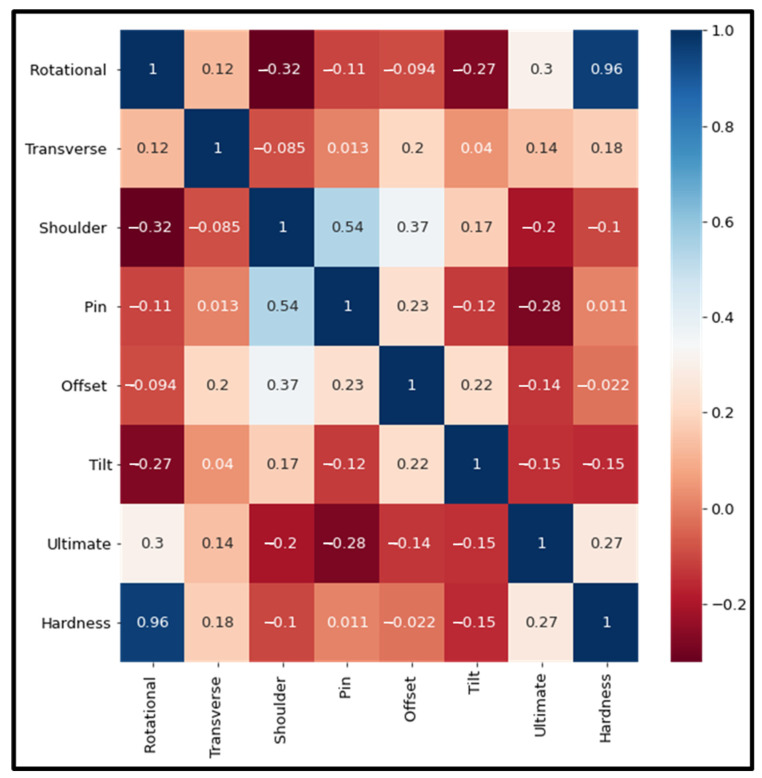
Correlation between the features of a dataset through a heat map.

Here, *x*_*i*_ is the new value of the sample, *y*_*i*_ represents the value of the training sample, and *i* is a sequential number of features. The decision tree (DT) is a supervised machine learning model that represents decision points as nodes, with each node branching out based on the results of sequential tests until a final decision is made. Random forest (RF) regression, illustrated in Figure 4, is an ensemble of decision trees built using a bagging algorithm. The trees in a random forest operate independently, and the individual predictions of each tree determine the final output of the RF model. One key advantage of the RF model is its ability to manage a diverse range of categorical and continuous variables. The predicted value $\hat{Y_{i}}$ is given by averaging the sum of individual trees, as per Equation (2) [39]:

(2) $$\hat{Y_{i}}=\frac{1}{N_{tree}}\sum_{n=1}^{N_{tree}}\hat{Y_{n}}$$

*N_tree_* is the total number of trees used in the random forest (RF). The RF model minimizes prediction error by aggregating the predictions of all decision trees within the random forest.
**Model****Training Parameters**LRRandom state = 42SVRKernel = ‘linear’, Random state = 40KNNN Neighbours = 2, Random state = 35DTCriterion = Mse, Random State = 10RFN estimators = 250, Random state = 0ANNNo. of hidden layer = 1, Neurons = 2–16

SVR is a regression algorithm derived from Support Vector Machines (SVM) principles. To enhance the generalization ability of learning machines by minimizing both structured risk and empirical risk, along with the confidence interval. Critical parameters in the SVR model include the kernel function type t, the penalty factor c, and the kernel function factor g. The radial basis function (RBF) is typically selected as the kernel function, which effectively addresses linearly non-separable problems through data transformation, as detailed in Equation (3). Additionally, t and c represent the tolerance for fitting errors in the model; a higher c increases the risk of overfitting. Meanwhile, g indicates the distribution of support vectors in the transformed feature space.

(3) $$k\left(x,\;y\right)=\exp\left(-g\times d{\left(x,\;y\right)}^{2}\right)$$

In order to effectively test and select a model, it is crucial to establish clear evaluation criteria. This will help in making informed decisions and choosing the best model for the task at hand. Here, we have used parameters such as coefficient of determination (COD, R^2^), mean square error (MSE), and mean absolute error (MAE), for selecting the most effective ML model for hardness and ultimate tensile strength estimation. Equations (4), (5), and (6) provide these parameters, respectively:

(4) $$MSE=\frac{1}{n}\sum_{i=1}^{n}\left(Y_{i}-Y^{\prime }\right)$$

(5) $$R^{2}=1-\frac{\sum_{i}\left(y_{i}-y^{\prime }\right)}{{\left(y_{i}-y_{mean}\right)}^{2}}$$

(6) $$MAE=\frac{\sum_{i=1}^{n}\left|y_{i}-\right.\left.y^{\prime }\right|}{n}$$

where

MSE = mean squared error

n = number of data points

Y_i_ = actual value

Y′ = predicted value

(7) $$Y_{\left(\mathrm{norm}\right)}=\frac{0.8*x}{\Delta }+(0.9-\frac{0.8*x\max}{\Delta })$$

Y_norm_ = normalized value

x = value to be normalized

$$\Delta =x_{max}-x_{min}$$

The model, which has a least mean squared error, mean average error and maximum coefficient of determination, was selected as the best model for further study, but before that, all the given data were normalized using Equation (7). All collected data (input and output data) were normalized between 0.1 and 0.9 [41]. To judge the accuracy of the model, the whole data set was split into two parts: one part for the training of the model (70% of the total data) and the other part for testing (30% of the entire data) using the train-test split function of Scikit learn library. Feature selection is a critical aspect of model generation in machine learning, as including irrelevant features can reduce the model’s accuracy [42]. Thus, it is essential to distinguish between significant and insignificant features (i.e., process parameters). To establish correlations among different features, the collected data were statistically analyzed using the Pearson correlation coefficient, calculated using the Python Pandas library [43]. The formula used to compute the correlation coefficient (Equation (8)) is provided below:

(8) $$r_{xy}=\frac{1}{n-1}\frac{\sum_{i=1}^{n}\left(xi-x^{\prime }\right)-\left(yi-y^{\prime }\right)}{\sigma_{x}\sigma_{y}}$$

where n is the sample size, *x*′ and *y*′ are the mean values of the two input features, and $\sigma_{y}$ and $\sigma_{y}$ are the standard deviations of the two features. The correlation coefficient value lies between −1 and 1, where 0 means no correlation, 1 means strong positive correlation, and −1 means strong negative correlation. The correlation coefficient values in the heat map diagram are shown in Figure 4.

Figure 4 shows a positive correlation between tool rotational speed, welding speed, tool tilt angle, and tool offset on UTS and hardness. Shoulder diameter and pin diameter have a negative correlation. However, the tool shoulder diameter positively correlates with the tool pin diameter. Therefore, all the features shown in the heat map were selected for the study.

**Figure 5:**
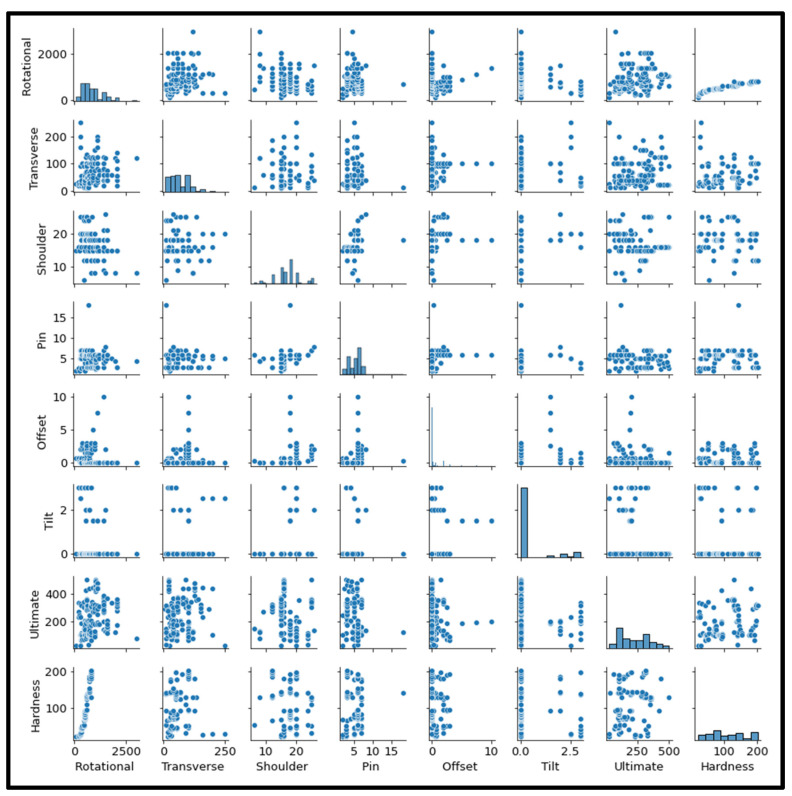
Correlation between the features of the dataset through scatterplot matrices.

Furthermore, to recognize the association between input and output features, scatter plots were plotted, as shown in Figure 5. The histogram shows the prediction of ultimate tensile strength and hardness for various friction stir welded plates based on multiple process parameters. It is also evident from Figure 5 that tool rotational speed, welding speed, shoulder diameter, tool pin diameter, tool offset, and tool tilt are very closely related, as their corresponding pair plots are very similar.

## 3. Experimental

**Figure 6:**
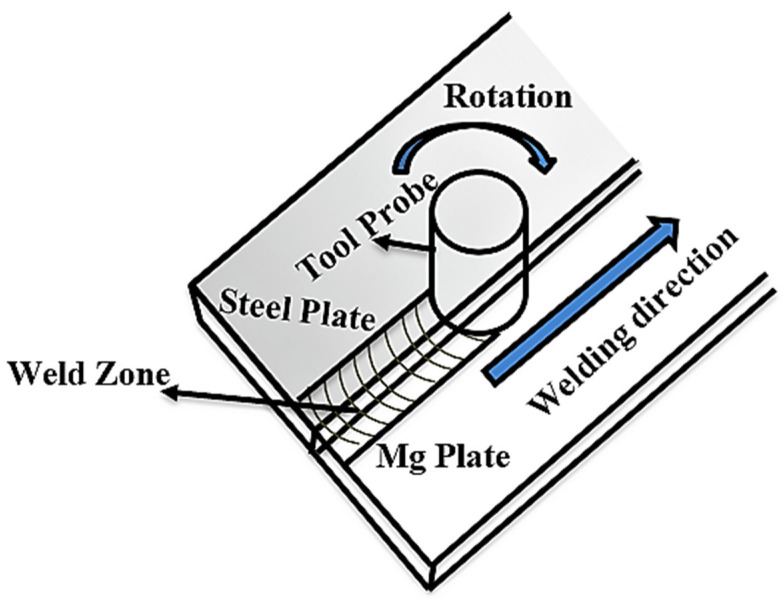
Schematic illustrations of the friction stir welding process.

For dissimilar material joining, the workpiece specimens were AZ61 magnesium alloys (Mg–5.8 wt. % percent Al–0.85 wt. % Zn) and low carbon steel plates with 200 mm × 50 mm × 1.5 mm dimensions were procured for experimentation and validation. The FSW was performed with a welding tool made of tungsten carbide (WC), with a 12 mm shoulder diameter, 4 mm probe diameter, and 1.3 mm probe length. The welding tool was set at a 3° tilt angle to the vertical axis. FSW was conducted in a butt configuration with a constant tool rotational speed of 1500 rpm. The tool traverse rates varied between 15, 30, and 50 mm/min. Figure 6 depicts the schematic arrangement of the friction stir welding (FSW) process for creating dissimilar joints.

An optical microscope was used for the metallographic studies. The OM specimens were manually polished using sand sheets of up to 4000 grits, followed by a final polish with 0.05 µm Al_2_O_3_ suspensions. To examine the microstructure and identify the elemental composition of the specimens, an SEM equipped with EDS was used.

## 4. Results and Discussion

### 4.1. Morphology of the Welded Joint

**Figure 7:**
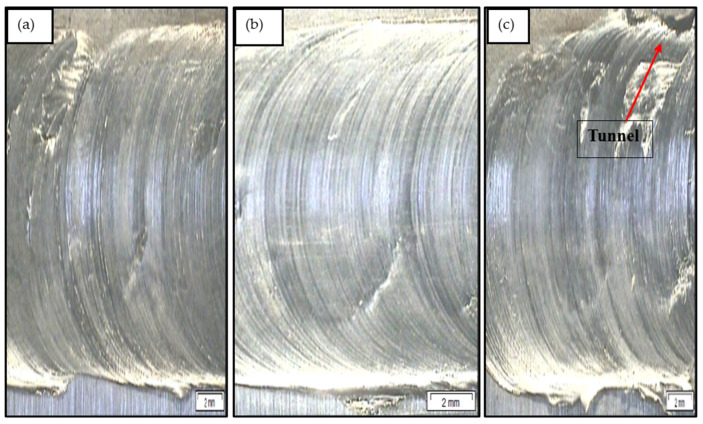
Surface appearance of dissimilar FSW joints between the AZ61 magnesium alloy and mild steel at a welding speed of (**a**) 15 mm/min, (**b**) 30 mm/min, and (**c**) 50 mm/min.

The surface appearance of all the welded specimens was good. Figure 7 depicts the changes in surface appearance with an increasing welding speed from 15 mm/min to 50 mm/min. The joint’s surface became smoother as the welding speed increased from 15 mm/min to 30 mm/min, as demonstrated in Figure 7a,b. Further increments in welding speed from 30 mm/min to 50 mm/min caused improper stirring of materials, which led to the slight appearance of tunnel defects. Proper mixing of materials was achieved at a welding speed of 30 mm/min, possibly due to the favourable working conditions. The proper mixing of materials promoted better mechanical interlocking with bending structures. Such interlocking results in a stronger joint and increased tensile strength. Finally, no flaws, such as pores, cavities, or tunnels, were observed at a greater scale in any of the joints.

### 4.2. Microstructure at the Interface

**Figure 8:**
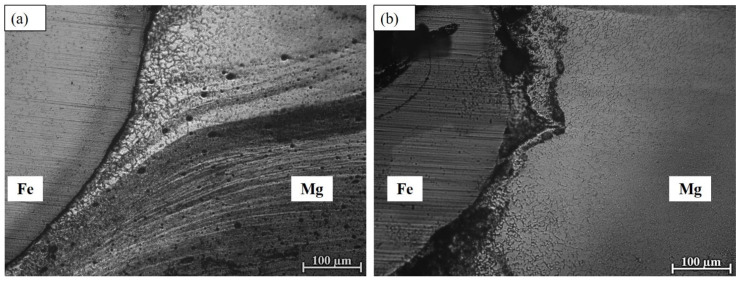
OM images of the interfaces of welded joints at a welding speed of (**a**) 30 mm/min and (**b**) 50 mm/min.

Figure 8 shows optical microscopic pictures of the cross-section interfaces at a tool rotation speed of 1500 rpm, traverse speed of 30 mm/min and 50 mm/min, and a tool offset location of 0.5 mm. The joint interface was uniform, with a 30 mm/min welding speed, as depicted in Figure 8a. However, with an increased welding speed, defects are observed at the joint interface, as shown in Figure 8b.

**Figure 9:**
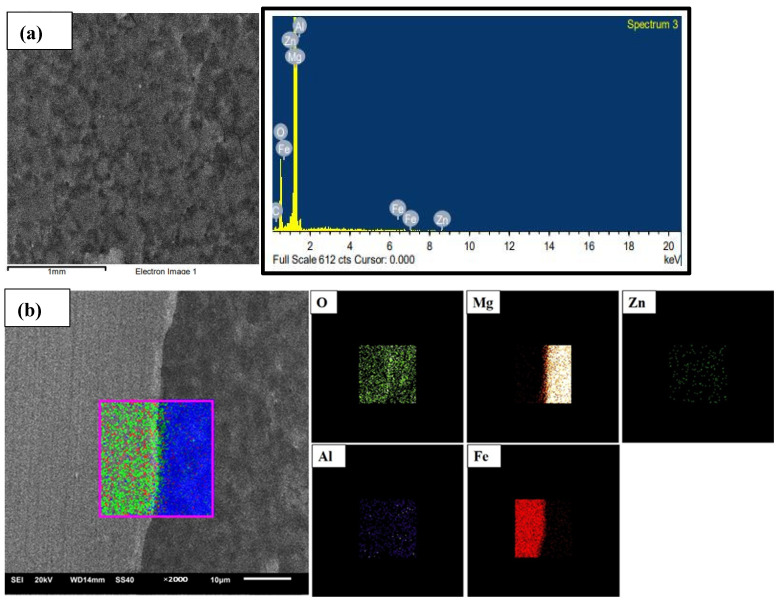
SEM image and EDS mappings of the interfaces on the cross-section of the specimen at 1500 rpm, 30 mm/min, offset 0.5 mm. (**a**) Point EDS at Mg side near-adjacent to interface. (**b**) EDS mapping of interface.

Figure 9 displays the SEM imaging and EDS mappings of the interfaces on the cross-section at a tool rotation speed of 1500 rpm, a welding speed of 30 mm/min, and a tool offset position of 0.5 mm. The EDS mapping indicates a region of aluminium depletion near the interface on the magnesium side compared to the aluminium content of the base AZ61 magnesium alloy, as shown in Figure 9a and 9b, respectively. Microstructural observations revealed the formation of an intermetallic compound of iron and aluminium at the joint interface due to the presence of aluminium in the magnesium alloy. Consequently, the Fe–Al intermetallic compound layer may form as the tool probe makes contact with the steel butt face, creating a new surface on the steel plate.

The aluminium in magnesium is thought to have been consumed during the production of the Fe–Al intermetallic compound.

## 5. Machine Learning Results

**Figure 10:**
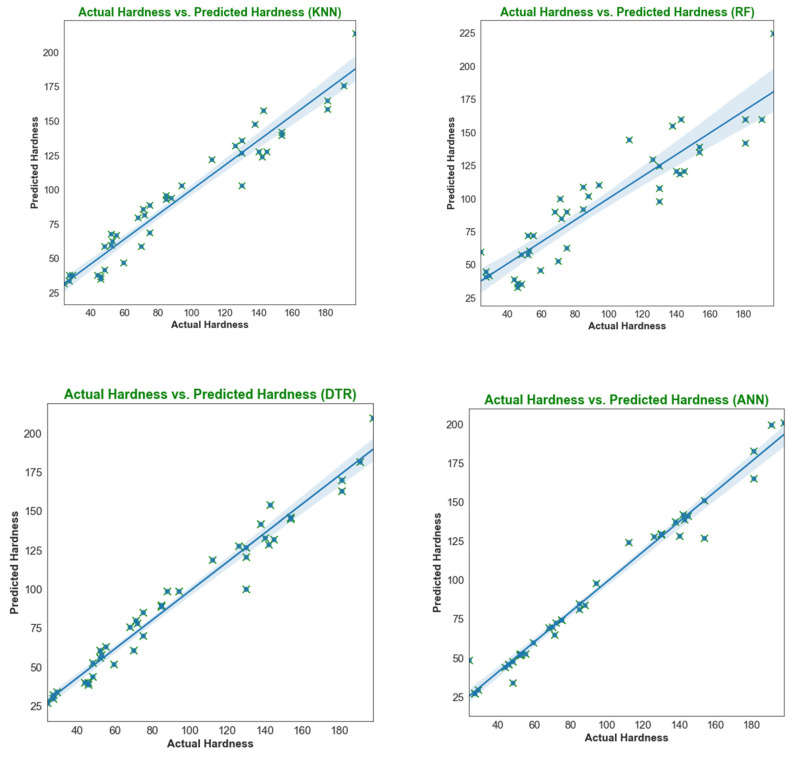
Training performance of LR, SVR, KNN, RF, DT, and ANN for hardness.

**Figure 11:**
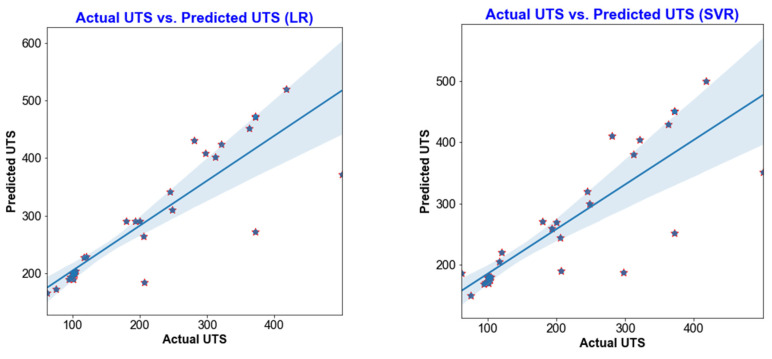

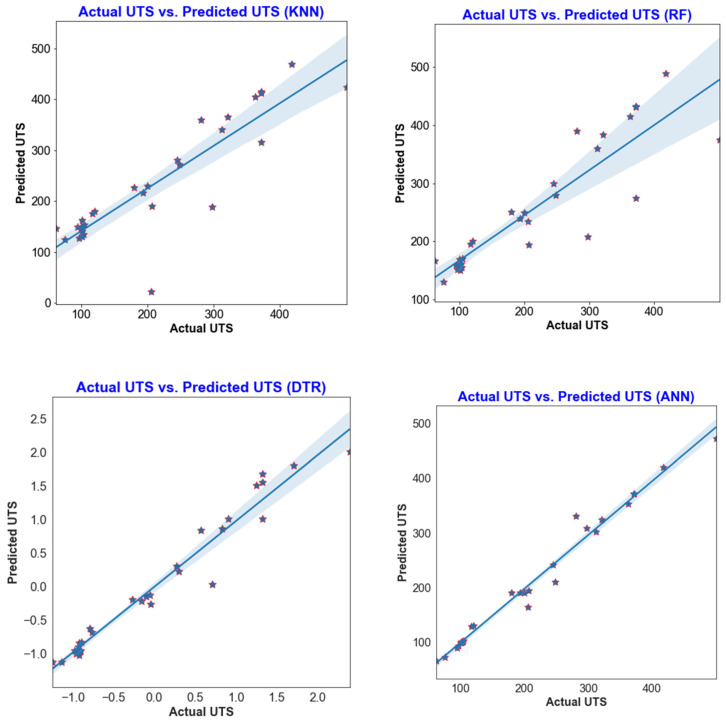
Training performance of LR, SVR, KNN, RF, DT, and ANN for UTS.

The results from testing the trained models on the remaining 30% of the dataset can be seen in Figure 10 and Figure 11. The output achieved from different machine learning (ML) models depends on the characteristics of the working dataset. An imbalance in the dataset impacts the model results, as is evident from the performance of different ML models. Figure 10 and Figure 11 illustrate that the estimation performance of ANN is significantly superior compared to LR, KNN, DT, SVR, and RF regression. The lower estimation accuracy of these models may be attributed to their simplicity. Furthermore, a simple, direct relationship is insufficient to describe the variation in hardness and ultimate tensile strength of FSW joints, likely due to the complex nature of the welding process. In this process, various parameters interact simultaneously, and even a slight change in any of them impacts the final output. Therefore, it is crucial to develop an effective machine learning model capable of accommodating the process’s non-linearity. The prediction results below indicate that the ANN model effectively achieved this objective.

Figure 10 and Figure 11 show that the artificial neural network (ANN) is the best fit for predicting both hardness and ultimate tensile strength (UTS). The consistent dispersion and variation of test data points around the regression line strongly indicate robust data fitting and the potential for highly accurate predictions in the ANN model.

**Figure 12:**
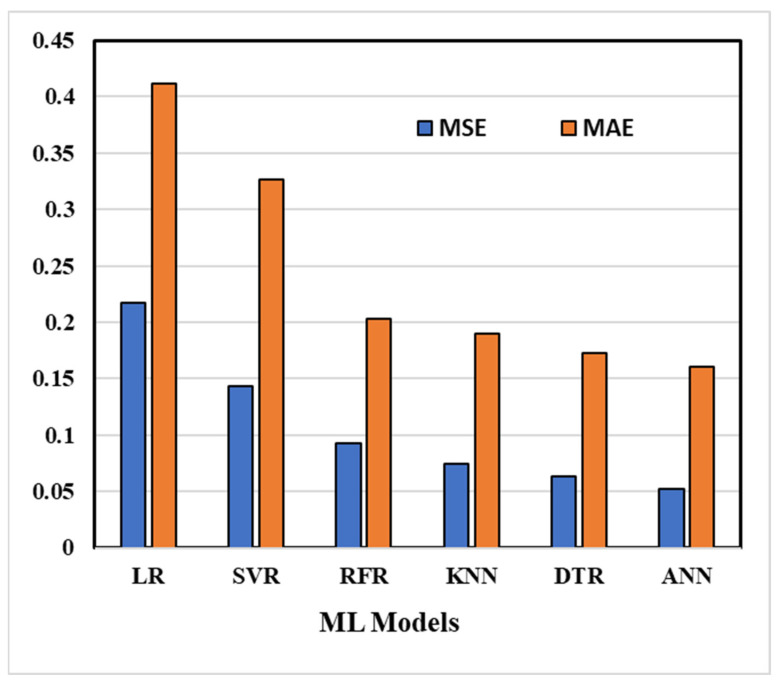
MSE and MAE for all six models under consideration.

**Figure 13:**
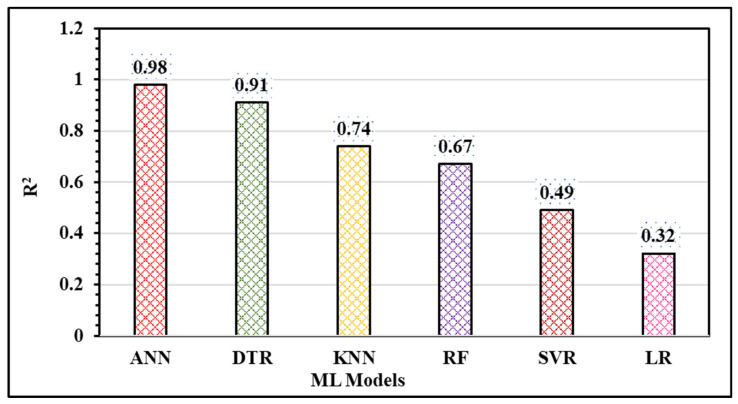
Correlation coefficient (R^2^) for all six models under consideration.

In Figure 12 and Figure 13, the MSE, MAE, and R^2^ values for all six machine learning models are displayed. The coefficient of determination (R^2^) value for the ANN model on the testing data is 0.91, which is notably higher than the values obtained for the other models. Moreover, the ANN model exhibits the highest R^2^ score and the lowest MAE and MSE (as depicted in Figure 13), suggesting that it is the most suitable method for accurately estimating hardness and UTS.

It has been seen that the ANN has the lowest mean squared error. Therefore, a feed forward back propagation artificial neural network was selected for further study.

### 5.1. ANN Modelling Using Python

Artificial neural networks (ANNs) are intelligent techniques inspired by biological neurons. Due to their finest qualities, such as generalization, faster processing, and simple implementation, ANNs have become quite popular in many engineering domains. ANNs are composed of several simple, interconnected processing units called neurons that are grouped into layers. Manufacturing, pattern identification, speech recognition, defect detection, communications, autonomous vehicles, and gantry crane navigation control are just a few sectors where artificial neural networks are widely used [43,44]. In manufacturing, ANNs are used in cold forging to forecast flow stress in hot deformation, tool wear monitoring, machining behaviour prediction, manufacturing process optimization, sintered density prediction, and many more [41,44]. In the training process, the network is provided with input and output pairs representing the pattern to be simulated, and the weights are determined using an iterative technique [45]. During the forward-back propagation network, each neuron in the hidden layer responds to each neuron in the output layer. In the backward pass, each hidden layer neuron unequivocally receives an error from the output layer through an activation function [45].

**Figure 14:**
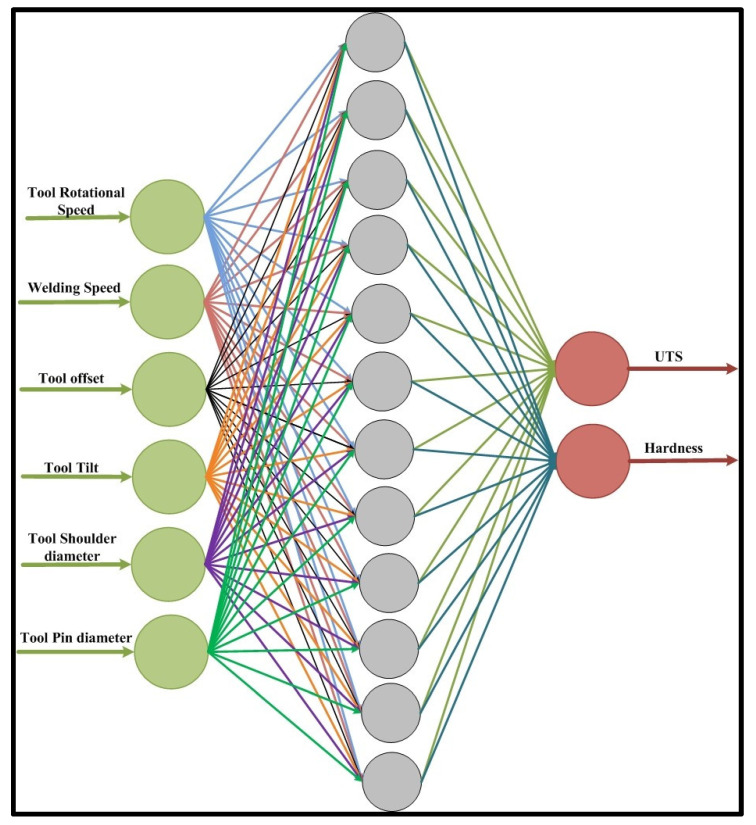
ANN architecture used for prediction of UTS and hardness.

In the present work, Python and its various libraries, such as Pandas, Scikit-learn, Numpy, Scipy, Matplotlib, etc., were utilized to create an ANN. Figure 14 depicts the topology of ANN that will be used to forecast the ultimate tensile strength and hardness for friction stir welded joints. Furthermore, the developed model is used to carry out the behavioural study of UTS and hardness with respect to different processing parameters. The feed-forward back propagation (B.P.) artificial neural network was first trained using 70% of the collected data from various kinds of literature, and then testing of the model was performed using 30% of the collected data that had not been utilized during training.

In this study, B.P. was employed with a single hidden layer, and ReLU was used as an activation function. Python and its various libraries were used to train and test the ANN model. The K-fold cross-validation technique (with 8 folds) was used to avoid overfitting the data. Furthermore, the output of the selected architecture was optimized by varying the number of neurons, keeping the limit to reduce the complexities of models. If the quantity of neurons is high in the hidden layer, more time and computational resources are utilized to generate satisfactory output.

**Figure 15:**
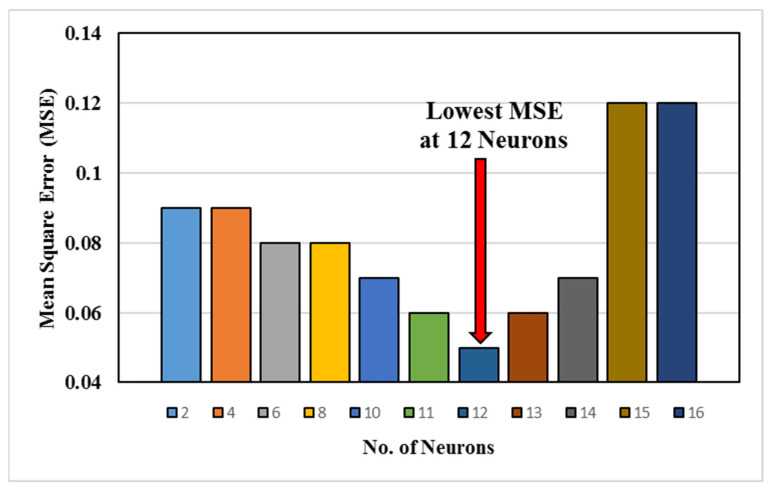
Variation in MSE with the number of neurons.

Figure 15 shows the change in the mean square deviation with the number of neurons. The selection of 12 neurons and a single hidden layer was based on a balance between model complexity and performance. During the preliminary testing phase, we varied the number of neurons and hidden layers to identify the configuration that minimized the mean squared error (MSE) while avoiding overfitting or excessive computational cost. Figure 15 in the manuscript shows the variation in MSE with the number of neurons, where the optimal performance was observed at 12 neurons. Increasing the number of neurons beyond this point resulted in diminishing returns in accuracy while increasing computational complexity.

A single hidden layer was chosen because it is sufficient to model the non-linear relationships between the input parameters (process parameters) and outputs (UTS and hardness) in this study. This is consistent with the universal approximation theorem, which states that a single hidden layer can approximate any continuous function, given sufficient neurons. Adding more layers did not significantly improve the accuracy, but increased training time and the risk of overfitting.

The training parameters used in this study are listed in Table 3. The first layer of ANN, the input layer, consists of process parameters, such as tool rotational speed, tool transverse speed, tool shoulder, pin diameter, tool offset, and tool tilt angle. The outer layer consists of UTS and hardness. When training of the defined network is completed, it is tested using well-defined test data. After the successful implementation of the training and testing, validation was performed. After successful validation, the described ANN model was used to predict the output behaviour, UTS, and hardness.

### 5.2. Prediction Using ANN

**Figure 16:**
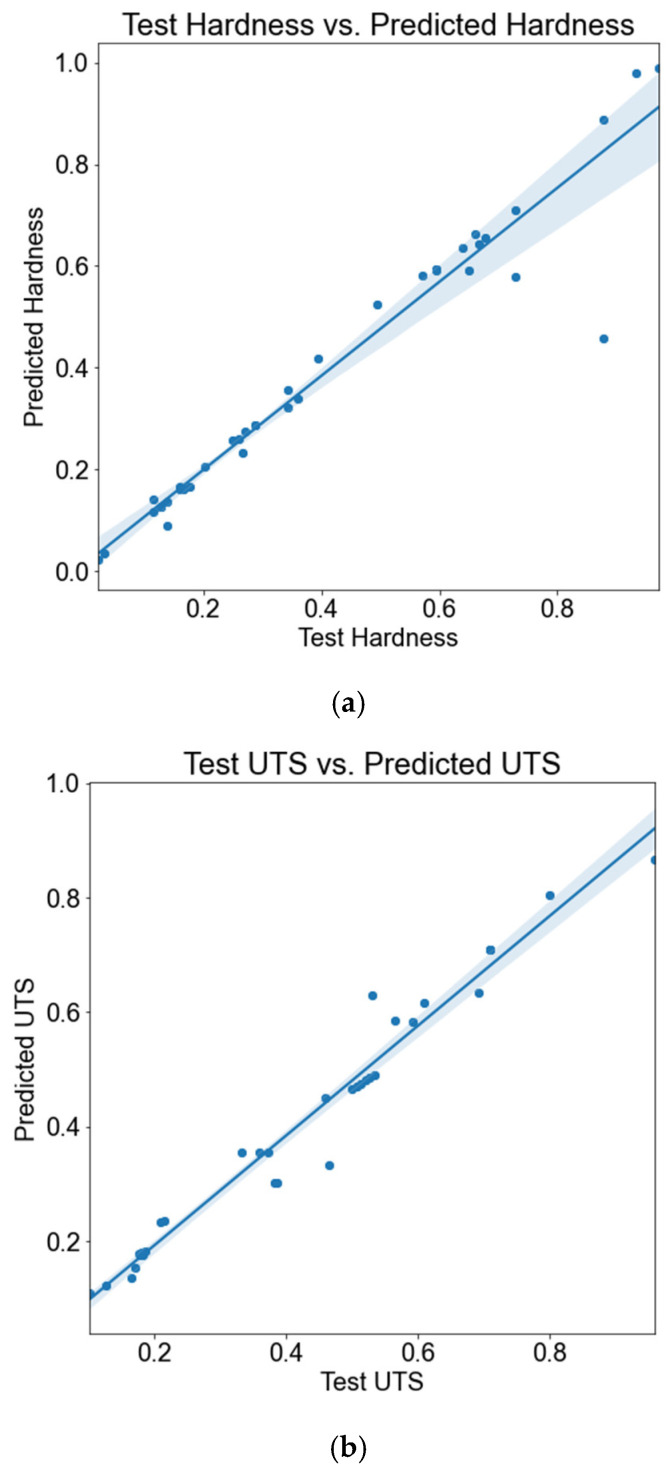
Graph between normalized test and predicted values of (**a**) hardness and (**b**) UTS.

Now, 30% of the total data, which were separated for testing, are used to test the generated model separately for both UTS and hardness. Figure 16 shows the graph between actual test hardness and UTS versus the corresponding predicted (normalized) values using ANN.

### 5.3. Behavioural Study of Predicted UTS and Hardness

**Figure 17:**
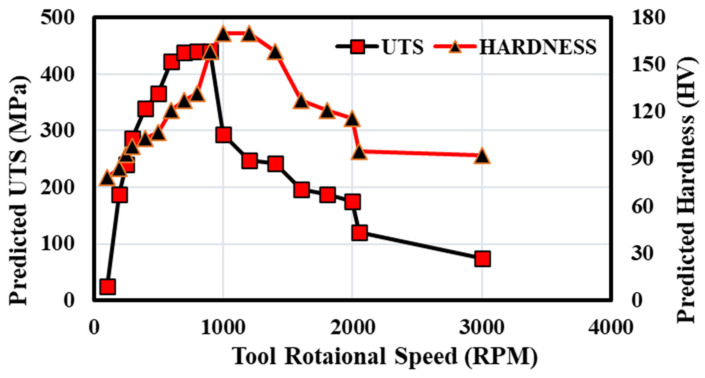
Variation in predicted UTS and hardness with tool rotational speed.

**Figure 18:**
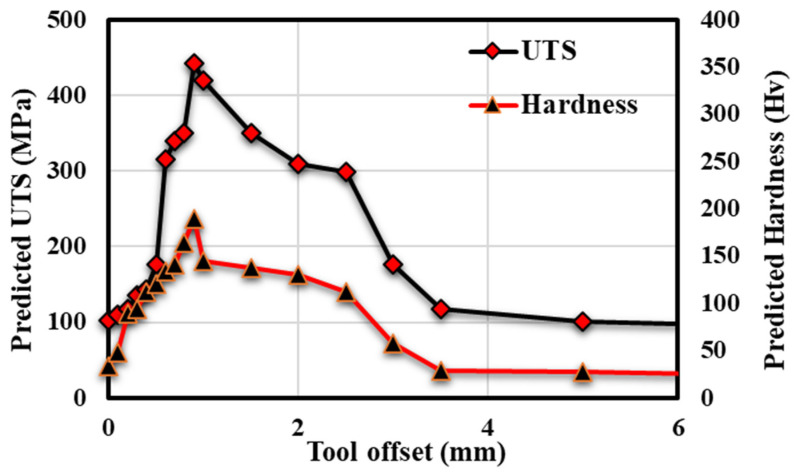
Variation in predicted UTS and hardness with tool offset.

After successfully validating the ANN model, further study was carried out in the present work to predict the behaviour of UTS and the hardness of different materials relative to different process parameters. Behavioural analysis was carried out by varying one of the process parameters at a time and keeping all other parameters constant. First, UTS and hardness were predicted by varying the tool rotational speed and keeping all other parameters constant. Secondly, tool offset was used as a varying parameter, keeping all other parameters constant. Figure 17 and Figure 18 show the predicted UTS and hardness behaviour by varying tool rotational speed and tool offset and keeping all other parameters constant.

The graph shows that both the UTS and hardness reach a maximum value and then decrease to a minimum value. Firstly, the value of predicted UTS and hardness by varying the tool rotational speed and tool offset increases and attains a maximum value, and then it starts decreasing and attains a minimum value. This result is similar to some of the previous experimental work performed by various researchers; the results of those experiments show a similar trend [46,47,48,49]. During FSW, tool rotation controls heat generation or input concerning the material’s plastic flow. The tool’s rotating speed affects the amount of plastic deformation and, as a result, material mixing. Extremely high rotation speeds result in excessive heat input, which causes a variety of flaws, such as flash, porosity, voids, and wormhole development. Low welding speeds result in more heat input and are linked to problems such as tunnelling [50]. Ugender et al. [51] observed that if the FSW tool rotational speed is below 900 rpm, pores will occur due to a lack of heat input and inadequate material filling. The tunnelling flaw was discovered at a rotational speed greater than 1400 rpm due to the heat generated. Various samples of 3 mm thick AA5052-O alloy were welded using FSW at tool rotation ranging from 800 to 3000 rpm while maintaining a tool traverse speed of 120 mm/min. The joint generated at a rotational speed of 1000 rpm had a UTS of 132 MPa, which was 74% compared to the base specimen [52]. Zafar et al., in their study, show the effect of tool rotational speed on FSW of ASTM A516-70 Steel using W–25%Re Alloy Tool. As the speed increases, both hardness and UTS increase, then decrease [53].

The studies by Nidhi et al. on the FSW of aluminium and copper and Yazdipour et al. on the FSW of dissimilar Al 5083-H321 and 316L stainless steel alloy joints show that by increasing the value of tool offset, UTS and hardness first increase and then decrease [54,55]. High-strength alloys typically require more heat to plasticize under FSW. As a result, an appropriate offset is required during dissimilar FSW. Because the varying thermal conductivity of dissimilar materials causes the heat distribution to be unbalanced in FSW, it can be adjusted by adding an offset. If a sufficient amount of tool offset is applied to the softer material (i.e., towards the harder material), the tool will be able to stir both materials [56] adequately. During the joining of dissimilar materials, an optimal tool offset becomes critical. A previous study demonstrated that welding with no pin offset produces poor weld quality with various flaws [57]. Watanabe et al. [58] analyzed the effect of tool offset on joining mild steel to aluminium alloy. With a tool pin offset of 0.2 mm toward the steel side, the highest strength (UTS) was achieved. Steel particles were broken into the aluminium matrix at a higher tool pin offset. These fragments were large enough to make holes, which weakened the weld’s tensile strength. Maintaining a pin offset that is too high or too low impairs weld quality and mechanical performance.

**Figure 19:**
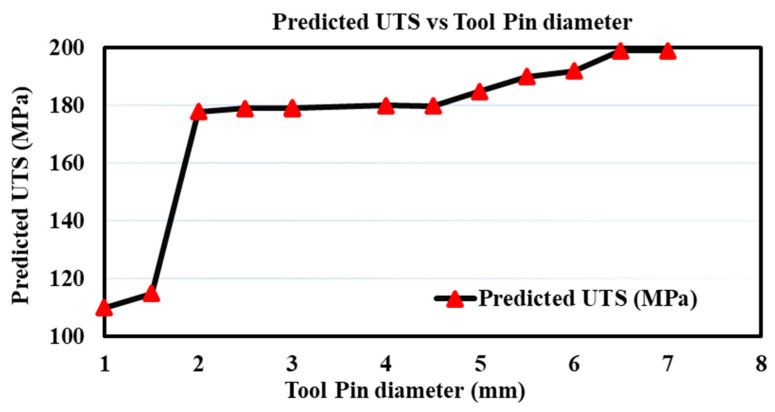
Variation in predicted UTS w.r.t. tool pin diameter.

It is visible from Figure 19 that the predicted UTS is closely related to tool pin diameter (in mm). The predicted UTS increases with an increase in tool pin diameter up to a particular value, and after that, it becomes constant. This result is similar to previous work done by Jadav et al. [15] and Anganan et al. [59,60,61].

### 5.4. The Experimental Results Prediction and Discussion

**Figure 20:**
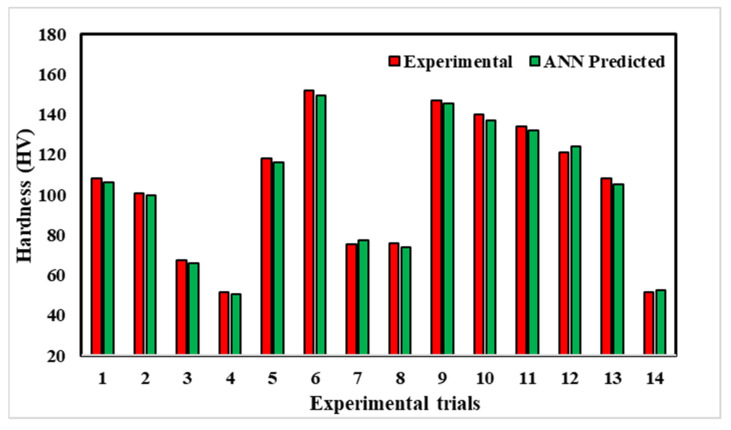
Comparison between experimental and modelling results for hardness by machine learning.

**Figure 21:**
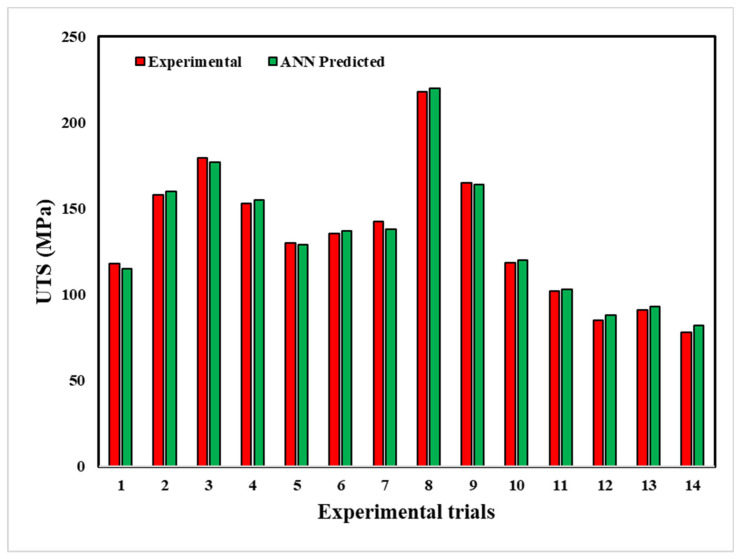
Comparison between experimental and modelling results for UTS by machine learning.

The ANN model predicted the hardness and ultimate tensile strength of the friction stir welded joints fabricated at different processing conditions, as shown in Figure 20 and Figure 21, respectively. The maximum and minimum hardness obtained is 152 HV and 51.3 HV, which differ by 1.5% and 2.3% from the value predicted by the ANN model. Furthermore, the maximum and minimum ultimate tensile strength obtained are 218 MPa and 78 MPa, which differ by 1.8% and 5.1% from the value predicted by the ANN model. Therefore, we can conclude that the experimental values agreed very well with the ANN-predicted values.

The prediction results of friction stir welded joints validate that the MLP method is very capable of dealing with nonlinear complex relations in material processing and property identification with high prediction accuracy. Therefore, we can easily switch to machine learning techniques over regression analysis, which is more suitable for linear problems. Furthermore, an abundance of data on friction stir welded joints is available in the literature, which can be used as raw data to improve and optimize joint properties and efficiencies using machine learning techniques. Additionally, the proposed ANN model can be extended to other welding processes, such as laser, arc, or resistance spot welding, by adjusting the input parameters specific to these methods. Similarly, it can be trained for diverse material combinations, including non-ferrous alloys and composites, to predict properties such as UTS and hardness. This adaptability highlights the model’s potential for broader applications in advanced manufacturing scenarios, such as aerospace and biomedical components.

## 6. Conclusions

In this paper, we applied six machine learning models to analyze process parameters affecting output responses, such as ultimate tensile strength and hardness, for FSW joints. The key findings are as follows:

- More than 200 groups of FSW data were collected, which include tool traverse speed, tool rotational speed, pin diameter, shoulder diameter, tool offset and tool tilt.
- The artificial neural network predicts the ultimate tensile strength and hardness with maximum accuracy among all trained and tested models. The ANN model with the 6-12-1 topology showed better testing and validation capability over attempted combinations.
- With the trial and tested machine learning model, the relationship between pin diameter, tool offset, and tool rotation speed over UTS and hardness was extracted from the entire range of collected data points.
- The hardness and ultimate tensile strength of the experimental results were predicted using the ANN model. The results obtained agree with the experimental data, with a maximum error of 2.9% for hardness and 5.1% for ultimate tensile strength. Therefore, it can be concluded that synthetic prediction by machine learning modelling can be used for various FSW joints to dramatically accelerate the discovery of strong and efficient joints.

### Future Research Directions

While the proposed models offer significant insights, further research is needed to enhance their applicability and reliability. Potential directions include:

- Expanding the dataset to include additional materials, joint configurations, and process conditions to improve the generalizability of the models.
- Exploring advanced machine learning techniques, such as ensemble models and deep learning architectures, for better predictive performance and feature interaction analysis.
- Investigating the influence of environmental conditions, such as temperature and humidity, on the welding process and incorporating them into predictive models.
- Integrating real-time monitoring and sensor data during the welding process for dynamic model updates and predictive maintenance.
- Applying the developed models in multi-objective optimization frameworks to simultaneously balance mechanical properties, cost, and process efficiency.

## 7. Practical Implications of the Findings

The developed machine learning models, particularly the ANN, provide a robust framework for understanding and predicting the effects of key process parameters on weld properties. These models can significantly aid welding design in the following ways:

1. Process Optimization: By accurately predicting ultimate tensile strength (UTS) and hardness, the models help identify optimal combinations of process parameters, reducing the need for extensive trial-and-error experimentation.
2. Defect Minimization: The insights derived from the models about the relationship between parameters (e.g., tool offset, rotational speed) and weld quality can guide the selection of parameter ranges to minimize defects such as voids or tunnels.
3. Material Pairing: The models are particularly valuable for dissimilar material welding, where parameter selection is crucial to balance heat distribution and mechanical properties, ensuring a strong and reliable joint.
4. Design for Applications: The predicted mechanical properties can inform the design of welded components tailored for specific applications, such as in the automotive and aerospace industries, where the strength-to-weight ratio and joint reliability are critical.

## Figures and Tables

**Table 1:** Collected and screened data of friction stir welded joints for machine learning.

S. No	Work Material	Rotational Speed (rpm)	Transverse Speed (mm/min)	Shoulder Diameter (D) mm	Pin Diameter (d) mm	Offset (mm)	Tilt Angle (°)	UTS (MPa)	Hardness
1	Similar	400	100	20	6	2	0	95	68
2	Similar	400	100	20	6	2.5	0	70	78
3	Similar	400	100	20	6	3	0	70	88
4	Similar	600	100	20	6	2	0	110	75
5	Similar	600	100	20	6	2.5	0	98	70
6	Similar	600	100	20	6	3	0	87	85
7	Similar	800	100	20	6	2	0	103	76
8	Similar	800	100	20	6	2.5	0	99	83
9	Similar	800	100	20	6	3	0	62	85
10	Similar	1000	100	20	6	2	0	103	76
11	Similar	1000	100	20	6	2.5	0	105	83
12	Similar	1000	100	20	6	3	0	87	175
13	Similar	100	25	15	2	0.6	0	25	191
14	Similar	250	25	15	2	0.2	0	240	203
15	Similar	500	25	15	2	0	0	100	202
16	Similar	1250	25	15	2	1	0	10	185
17	Similar	280	160	20	5	0.4	2.5	230	180
18	Similar	280	200	20	5	0	2.5	100	194
19	Similar	280	250	20	5	0.4	2.5	25	198
20	Similar	280	315	20	5	0	2.5	0	197
21	Similar	1500	40	26	8	2	2	133	181
22	Similar	560	69	18	6	0.2	2	200	89
23	Similar	560	69	18	6	0.3	2	191	88
24	Similar	710	69	18	6	0.6	2	207	85
25	Similar	710	69	18	6	0.3	2	198	88
26	Similar	800	75	18	6	0	0	175	68
27	Similar	1000	75	18	6	0	0	177	78
28	Similar	1200	75	18	6	0	0	180	88
29	Similar	1400	75	18	6	0	0	140	75
30	Similar	1600	75	18	6	0	0	140	70
31	Similar	300	90	25	7	2	0	330	112
32	Similar	450	90	25	7	2	0	340	125
33	Similar	600	90	25	7	2	0	350	130
34	Similar	850	90	25	7	2	0	340	133
35	Similar	600	50	25	7	2	0	360	130
36	Similar	600	70	25	7	2	0	350	130
37	Similar	600	132	25	7	2	0	350	130
38	Similar	600	90	25	7	0.5	0	360	130
39	Similar	600	90	25	7	1	0	350	130
40	Similar	600	90	25	7	1.5	0	500	130
41	Similar	600	90	25	7	2.5	0	300	130
42	Similar	500	50	20	4	0	3	200	85
43	Similar	630	50	20	4	0	3	197	76
44	Similar	800	50	20	4	0	3	198	83
45	Similar	500	50	20	4	1.5	3	206	85
46	Similar	630	50	20	4	1.5	3	203	76
47	Similar	800	50	20	4	1.5	3	204	83
48	Similar	900	60	15	5	0	0	270	175
49	Similar	1200	60	15	5	0	0	310	191
50	Similar	1400	60	15	5	0	0	372	203
51	Similar	1600	60	15	5	0	0	367	202
52	Similar	1800	60	15	5	0	0	334	185
53	Similar	1400	20	15	5	0	0	280	180
54	Similar	1400	40	15	5	0	0	363	194
55	Similar	1400	60	15	5	0	0	372	198
56	Similar	1400	80	15	5	0	0	371	197
57	Similar	1400	100	15	5	0	0	261	179
58	Similar	1400	60	9	5	0	0	266	178
59	Similar	1400	60	12	5	0	0	310	193
60	Similar	1400	60	15	5	0	0	372	198
61	Similar	1400	60	18	5	0	0	271	197
62	Similar	1400	60	21	5	0	0	350	187
63	Similar	1400	60	15	3	0	0	281	181
64	Similar	1400	60	15	4	0	0	331	194
65	Similar	1400	60	15	5	0	0	372	198
66	Similar	1400	60	15	6	0	0	340	197
67	Similar	1400	60	15	7	0	0	321	178
68	Similar	224	20	16	2.8	0.7	3	228	140
69	Similar	224	25	16	2.8	0.7	3	270	145
70	Similar	224	31.5	16	2.8	0.7	3	332	143
71	Similar	355	20	16	2.8	0.7	3	116	144
72	Similar	355	25	16	2.8	0.7	3	182	142
73	Similar	355	31.5	16	2.8	0.7	3	312	140
74	Similar	450	31.5	16	2.8	0.7	3	70	138
75	Similar	450	12	24	6	0.3	0	112	134
76	Similar	560	12	24	6	0.3	0	76	130
77	Similar	700	12	24	6	0.3	0	28	126
78	Similar	700	20	24	6	0.3	0	97	122
79	Similar	700	30	24	6	0.3	0	100	118
80	Similar	700	12	18	18	0.3	0	118	114
81	Similar	450	12	6	6	0.3	0	146	110
82	Similar	2050	20	6	4	0	0	275	106
83	Similar	2050	40	8	4	0	0	300	102
84	Similar	2050	56	12	6	0	0	310	98
85	Similar	710	40	15	3	0	0	245	186
86	Similar	710	50	15	3	0	0	290	145
87	Similar	900	31.5	15	3	0	0	268	154
88	Similar	900	40	15	3	0	0	270	146
89	Similar	900	50	15	3	0	0	322	181
90	Similar	1120	31.5	15	3	0	0	180	143
91	Similar	1120	40	15	3	0	0	248	142
92	Similar	1120	50	15	3	0	0	312	126
93	Similar	635	8	15	6	0	0	290	135
94	Similar	710	28	18	6	0	0	166	142
95	Similar	500	40	18	6	0	0	136	126
96	Similar	500	40	18	6	0	0	132	135
97	Similar	355	40	18	5	0	0	69	140
98	Similar	500	28	18	7	0	0	156	142
99	Similar	355	56	18	6	0	0	64	126
100	Similar	500	56	18	5	0	0	109	135
101	Dissimilar	500	56	18	7	0	0	122	140
102	Dissimilar	500	28	18	6	0	0	92	142
103	Dissimilar	355	56	18	6	0	0	95	126
104	Dissimilar	710	40	18	6	0	0	140	135
105	Dissimilar	500	40	18	6	0	0	131	140
106	Dissimilar	500	40	18	5	0	0	123	142
107	Dissimilar	710	40	18	7	0	0	112	126
108	Dissimilar	500	28	18	5	0	0	164	135
109	Dissimilar	500	40	18	6	0	0	143	140
110	Dissimilar	355	40	18	7	0	0	64	142
111	Dissimilar	500	40	20	6	0	0	102	126
112	Dissimilar	355	28	20	5	0	0	99	135
113	Dissimilar	710	40	20	6	0	0	104	140
114	Dissimilar	710	56	20	7	0	0	105	142
115	Dissimilar	710	28	20	5	0	0	103	126
116	Dissimilar	500	40	20	6	0	0	102	135
117	Dissimilar	355	28	20	7	0	0	100	140
118	Dissimilar	500	40	20	7	0	0	102	142
119	Dissimilar	500	40	20	5	0	0	101	126
120	Dissimilar	355	56	20	5	0	0	100	135
121	Dissimilar	500	56	20	6	0	0	103	140
122	Dissimilar	710	28	20	7	0	0	104	142
123	Dissimilar	500	40	20	6	0	0	102	126
124	Dissimilar	355	56	20	7	0	0	101	135
125	Dissimilar	710	56	20	5	0	0	105	140
126	Dissimilar	500	40	20	6	0	0	102	142
127	Dissimilar	500	40	20	6	0	0	102	126
128	Dissimilar	500	28	20	6	0	0	102	135
129	Dissimilar	500	40	20	6	0	0	102	140
130	Dissimilar	355	40	20	6	0	0	100	142
131	Dissimilar	1600	40.2	15	6	0	0	117	71
132	Dissimilar	1600	40.2	18	6	0	0	205	75
133	Dissimilar	1600	40.2	21	6	0	0	197	72
134	Dissimilar	1600	40.2	18	6	0	0	162	72
135	Dissimilar	1600	40.2	18	6	0	0	160	73
136	Dissimilar	1600	40.2	18	6	0	0	159	73
137	Dissimilar	1600	40.2	18	6	0	0	207	77
138	Dissimilar	1600	40.2	18	6	0	0	178	75
139	Dissimilar	1120	80	20	5.7	0	0	323	96
140	Dissimilar	1120	100	20	5.7	0	0	371	93
141	Dissimilar	1120	125	20	5.7	0	0	341	94
142	Dissimilar	1120	160	20	5.7	0	0	432	96
143	Dissimilar	710	20	20	4.5	0	0	150	78
144	Dissimilar	710	28	20	4.5	0	0	151	9
145	Dissimilar	710	40	20	4.5	0	0	100	63
146	Dissimilar	1000	20	20	4.5	0	0	100	92
147	Dissimilar	1000	28	20	4.5	0	0	160	13
148	Dissimilar	1000	40	20	4.5	0	0	125	16
149	Dissimilar	1400	20	20	4.5	0	0	130	80
150	Dissimilar	1400	28	20	4.5	0	0	90	4
151	Dissimilar	1400	40	20	4.5	0	0	125	16
152	Dissimilar	600	50	12	3	0	0	312	36
153	Dissimilar	1000	150	12	3	0	0	299	42
154	Dissimilar	600	50	12	3	0	0	315	27
155	Dissimilar	1000	150	12	3	0	0	272	30
156	Dissimilar	600	50	12	3	0	0	281	48
157	Dissimilar	1000	150	12	3	0	0	318	52
158	Dissimilar	600	50	12	3	0	0	282	44
159	Dissimilar	1000	150	12	3	0	0	293	48
160	Dissimilar	463	16	12	3	0	0	302	34
161	Dissimilar	1136	184	12	3	0	0	291	48
162	Dissimilar	800	100	12	3	0	0	286	56
163	Dissimilar	800	100	12	3	0	0	299	32
164	Dissimilar	800	100	12	3	0	0	298	29
165	Dissimilar	800	100	12	3	0	0	319	52
166	Dissimilar	800	100	12	3	0	0	318	46
167	Dissimilar	800	100	12	3	0	0	315	46
168	Dissimilar	800	100	12	3	0	0	315	46
169	Dissimilar	800	100	12	3	0	0	315	46
170	Dissimilar	800	100	12	3	0	0	313	46
171	Dissimilar	800	100	12	3	0	0	310	46
172	Dissimilar	560	125	16	5.7	0	0	423	152
173	Dissimilar	710	125	16	5.7	0	0	439	144
174	Dissimilar	900	125	16	5.7	0	0	442	126
175	Dissimilar	1120	160	16	5.7	0	0	445	70
176	Dissimilar	1120	200	16	5.7	0	0	435	182
177	Dissimilar	750	100	20	6	0	2	105	154
178	Dissimilar	750	100	20	6	0.5	2	139	143
179	Dissimilar	750	100	20	6	1	2	150	132
180	Dissimilar	750	100	20	6	1.5	2	134	112
181	Dissimilar	1000	15	12	4	1	0	115	90
182	Dissimilar	1500	15	12	4	1	0	160	138
183	Dissimilar	2000	15	12	4	1	0	180	147
184	Dissimilar	2500	15	12	4	1	0	155	121
185	Dissimilar	3000	15	12	4	1	0	129	105
186	Dissimilar	1000	30	12	4	1	0	137	111
187	Dissimilar	1500	30	12	4	1	0	138	119
188	Dissimilar	2000	30	12	4	1	0	222	153
189	Dissimilar	2500	30	12	4	1	0	165	145
190	Dissimilar	3000	30	12	4	1	0	121	101
191	Dissimilar	1000	50	12	4	1	0	103	85
192	Dissimilar	1500	50	12	4	1	0	90	70
193	Dissimilar	2000	50	12	4	1	0	94	75
194	Dissimilar	2500	50	12	4	1	0	82	55
195	Dissimilar	3000	50	12	4	1	0	59	42
196	Dissimilar	1000	50	12	4	0	0	290	155
197	Dissimilar	1500	50	12	4	0	0	325	170
198	Dissimilar	2000	50	12	4	0	0	495	220
199	Dissimilar	2500	50	12	4	0	0	418	200
200	Dissimilar	3000	50	12	4	0	0	372	190

**Table 2:** Various groups along with parameters.

Processing Parameters	Tool rotational speed	RPM
	Tool transverse speed	mm/min
	Tool shoulder diameter	mm
	Tool Pin diameter	mm
	Tool offset	mm
	Tool tilt angle	degree (°)

**Table 3:** Model training parameters.

1	Network Topology	6-12-1
2	Total hidden layer	1
3	Total hidden neuron	12
4	Data used for training	70%
5	Data used for testing	30%
6	Number of epochs	1000
7	Learning rate	0.01

## Data Availability

The original contributions presented in the study are included in the article, further inquiries can be directed to the corresponding author.
